# Chloroquine Resistant *Plasmodium vivax*: *In Vitro* Characterisation and Association with Molecular Polymorphisms

**DOI:** 10.1371/journal.pone.0001089

**Published:** 2007-10-31

**Authors:** Rossarin Suwanarusk, Bruce Russell, Marina Chavchich, Ferryanto Chalfein, Enny Kenangalem, Varakorn Kosaisavee, Budi Prasetyorini, Kim A. Piera, Marion Barends, Alan Brockman, Usa Lek-Uthai, Nicholas M. Anstey, Emiliana Tjitra, François Nosten, Qin Cheng, Ric N. Price

**Affiliations:** 1 International Health Program, Infectious Diseases Division, Menzies School of Health Research and Charles Darwin University, Darwin, Australia; 2 Department of Drug Resistance and Diagnostics, Australian Army Malaria Institute, Brisbane, Australia; 3 National Institute of Health Research and Development and Menzies School of Health Malaria Research Program, Timika, Indonesia; 4 District Ministry of Health, Timika, Papua, Indonesia; 5 Department of Parasitology, Faculty of Public Health, Mahidol University, Bangkok, Thailand; 6 National Institute of Health Research and Development, Ministry of Health, Jakarta, Indonesia; 7 Faculty of Tropical Medicine, Mahidol University, Bangkok Thailand; 8 Shoklo Malaria Research Unit, Mae Sod, Tak Province, Thailand; 9 Centre for Vaccinology and Tropical Medicine, Nuffield Department of Clinical Medicine, John Radcliffe Hospital, Oxford, United Kingdom; London School of Hygiene & Tropical Medicine, United Kingdom

## Abstract

**Background:**

Treatment failure of chloroquine for *P. vivax* infections has reached high levels in the eastern provinces of Indonesia, however, *in vitro* characterization of chloroquine resistance and its associated molecular profile have yet to be determined.

**Methods:**

Using a modified schizont maturation assay we investigated the *in vitro* chloroquine susceptibility profile and molecular polymorphisms of *P. vivax* isolates collected from Papua, Indonesia, where high levels of clinical chloroquine treatment failure have been reported, and from Thailand, where chloroquine treatment is generally effective.

**Results:**

The geometric mean chloroquine IC_50_ for *P. vivax* isolates from Papua (n = 145) was 312 nM [95%CI: 237–411 nM] compared to 46.8 nM [95%CI: 34.7–63.1 nM] from Thailand (n = 81); p<0.001. Correlating with the known clinical efficacy of the area, a cut off for chloroquine resistance was defined as 220nM, a level exceeded in 13.6% (11/81) of Thai isolates and 65% (94/145) of Papuan isolates; p<0.001. Several sequence polymorphisms in *pvcrt-o* and *pvmdr1*, and difference in pvmdr1 copy number were identified. A Y976F mutation in *pvmdr1* was present in 96% (123/128) of Papuan isolates and 25% (17/69) of Thai isolates; p<0.001. Overall, the geometric mean chloroquine IC_50_ in isolates with the Y976F mutation was 283 nM [95%CI: 211–379], compared to 44.5 nM [95%CI: 31.3–63.4] in isolates with the wild type; *p*< 0.001. *Pvmdr1* amplification occurred in 23% (15/66) of Thai isolates compared to none (0/104) of Indonesian isolates (p<0.001), but was not associated with increased chloroquine resistance after controlling for geographical location.

**Conclusions:**

*In vitro* susceptibility testing of *P. vivax* discriminates between populations with differing levels of clinical efficacy of chloroquine. The *pvmdr1* polymorphism at Y976F may provide a useful tool to highlight areas of emerging chloroquine resistance, although further studies defining its clinical correlates are needed.

## Introduction

The burden of malaria caused by *Plasmodium vivax* has been greatly under-appreciated both in terms of its clinical spectrum and incidence of disease [1], [2]. *P. vivax* is the most widely distributed cause of malaria in the world affecting 40% of the worlds population and causing between 147–436 million clinical infections each year [3]. Although associated with less mortality than *P falciparum* it exerts a considerable morbidity particularly in children and pregnant women. Control measures are confounded by two major factors: firstly, the presence of dormant hypnozoite stages in the liver, which result in relapse infections weeks after the cure of the initial episode, and secondly the emergence of chloroquine resistance.

In most of the world chloroquine remains the first line of treatment for patients with vivax malaria. Not only is it well-tolerated and affordable, but its long half-life provides protection from early relapses following treatment. The first cases of chloroquine resistant *P. vivax* were reported in 1989 from PNG [4] and northern Papua (formerly Irian Jaya), Indonesia [5], [6], [7], [8]. Chloroquine monotherapy is now virtually ineffective in Papua Indonesia [8], [9], [10] with significant clinical resistance apparent throughout the Indonesian archipelago [5], [11]. More recently sporadic cases have been reported from Myanmar [12], South America [13], [14], Viet Nam [15], and Turkey [16].

Despite these clinical reports, the global prevalence of chloroquine resistant *P. vivax* remains poorly defined. Clinical studies are difficult to carry out and subject to individual variations in patient immune status, reinfection and frequent relapses. *In vitro* susceptibility assays provide an alternative means of assessing drug susceptibility of *Plasmodium* spp. Although these tests have been well established for *P. falciparum*, their application in *P. vivax* has been more difficult to develop due to limitations of in vitro culture methods in this species. Recently several centres have reported methods for conducting *in vitro P. vivax* drug susceptibility which are generally based on the *P. falciparum* WHO microtest using quantification of schizont maturation [17], [18], [19].

The mechanism of *P. vivax* chloroquine resistance is unknown and as yet no genetic markers have been identified. In *P. falciparum*, polymorphisms in *pfcrt* and *pfmdr1* have been shown to confer resistance [20], [21]. However, no associations have been found between point mutations in the orthologue genes, *pvcrt-o* (*pvcg10*) and *pvmdr1* and the clinical response of vivax malaria to chloroquine [22], [23]. Heterologous systems investigating the effect of *pvcrt-o* expression on chloroquine response showed a 2.2-fold decrease in susceptibility to chloroquine in *P. falciparum* transformed with *pvcrt-o,* suggesting a possible role of *pvcrt-o* in chloroquine resistant *P. vivax* [24].

In this study, we investigated the *in vitro* chloroquine susceptibility profile and molecular polymorphisms of *P. vivax* isolates collected from Papua, Indonesia, where high levels of clinical chloroquine resistance have been reported [10], [25] and from Thailand where chloroquine treatment is generally effective [26], [27].

## Materials and Methods

### Field location and sample collection

Clinical isolates were collected between 2003 and 2006 from two sites, one in Indonesia and the other in Thailand. Timika, located in the southern region of Papua Province, Indonesia, has documented clinical chloroquine resistance with day 28 failure rates following chloroquine monotherapy exceeding 65% and 16% of patients having early treatment failure [10]. At the second site at the Shoklo Malaria Research Unit, Mae Sod, Tak Province on the western border of Thailand, *P. vivax* remains clinically sensitive to chloroquine [27].

Patients with symptomatic infections of pure *P. vivax* presenting to an outpatient facility were recruited into the study and 5 ml blood samples collected by venepuncture. After removal of host white blood cells using a CF11 column, 2 ml of packed infected Red blood cells (IRBC) were divided as follows: 1 ml was cryopreserved in glycerolyte, 200 µl spotted onto a filter paper and 800 µl was used for the *in vitro* drug susceptibility assay. Patients were treated with dihydroartemisinin-piperaquine (Indonesia) or chloroquine (Thailand) according to local guidelines, but were not followed routinely thereafter.

### *In vitro* drug susceptibility assay

*P. vivax* susceptibility to chloroquine was measured at both sites using an identical protocol modified from the WHO microtest as described previously [17]. This method was modified further by reducing the final haematocrit of the blood media mix (BMM) from 4% to 2%; and using 200 µl of BMM per well instead of 50 µl. The BMM were added to pre-dosed drug plates containing serial concentrations of chloroquine with doubling dilutions from 2992 nM to 2.92 nM (Salt). Drug plates were quality assured using *P. falciparum* clones with known chloroquine susceptibility: the resistant clones K1 and W2 had median chloroquine IC_50s_ of 120 nM and 470nM respectively compared to the chloroquine sensitive clones 3D7 and FC27 with median IC_50s_ of 17 nM and 9 nM.

A traditional candle jar was used to mature the parasites at 37.5°C (25–36 hours) at reduced oxygen concentration. Incubation was stopped when parasites present had matured to at least 40% schizonts in the drug-free control well. A thick blood film was made from each well, stained with Giemsa and examined microscopically. The number of schizonts per 200 asexual stage parasites was determined and the result for each drug concentration normalized to the control well. The dose-response data were analyzed using nonlinear regression analysis (WinNonLin 4.1, Pharsight Corperation) to obtain the IC_50_ values. To assess the effect of verapamil on chloroquine susceptibility, an additional assay was conducted on 16 Indonesian isolates in which 0.9 µM verapamil was added to serial dilutions of chloroquine and the IC50 calculated and compared to that of chloroquine alone.

### Malaria DNA preparation and determinantion of species and hapltotype

Genomic DNA from blood spots and cryopreserved samples was extracted using QIAamp DNA mini kit (Qiagen). *Plasmodium* species were confirmed using multiplex PCR as previously described [28]. The haplotypes of samples containing a single *P. vivax* infection were then determined using three polymorphic markers, by sequencing *pvama1* [29], the number of *pvmsp1* bands after PCR [29] and restriction fragment polymorphism of the *pvmsp3* alpha locus [30].

### SNP identification in *pvmdr1* and *pvcrt-o* genes

In order to identify relevant polymorphisms in the *pvmdr1* and *pvcrt-o* genes in our parasite population 25 Indonesian and 7 Thai *P. vivax* isolates (“core” samples), were fully sequenced for both genes using primers listed in Table 1, comparing the sequences to those of the *pvmdr1* (GenBank Acc. No. AY618622) and *pvcrt-o* (GenBank Acc. No. AF314649) of the Sal 1, a chloroquine sensitive strain from Salvador used as a reference strain in this study. All core isolates were single species, monoclonal infections. PCR conditions were as follows: a total volume of 50 µl containing 5 µl of 10×PCR buffer, 2.5 mM MgCl_2_, 0.20 mM each dNTP, 1 µM each primer and 1.25 U of AmpliTaq Gold DNA polymerase (Applied Biosystems), and 1 µl of genomic DNA. PCR was performed under the following conditions: 95°C for 10 minutes and 40 cycles of at 94°C for 40 seconds, 55°C for 1 minutes and 72°C for 2 minutes. PCR products were sequenced using the BigDye terminator 3.1 (Applied Biosystems). Polymorphisms which were identified in the core samples were then examined in the complete sample set.

**Table 1:** Primers and sequences used to study mutations in *pvmdr1* and *pvcrt-o* and in the pvmdr1 copy number assay.

**A. ** ***pvmdr1***	Pvmdr1-1F	5′-CTT TTA TGC CTC TCC CCC
	Pvmdr1-1Fb	5′-AGA TTG TTC TGT AGC CGTT
	Pvmdr1-1R	5′-GCG TAA GAT GCT AAA ATG AACC
	Pvmdr1-2F	5′-ATT TAA CCT TTC AGA AAA GCT GT
	Pvmdr1-2R	5′-CCA CCT GAC AAC TTA GAT GC
	Pvmdr1-3F	5′-CTG ATA CAA GTG AGG AAG AAC TAC
	Pvmdr1-3R	5′-ACT ATC CTG GTC AAA AAA GC
	Pvmdr1-4F	5′-CCC TCT ACA TCT TAG TCA TCG
	Pvmdr1-4R	5′-TGG TCT GGA CAA GTA TCT AAAA
	Pvmdr1-5F	5′-GGA AGT TGA TGT CCC TAA AGG
	Pvmdr1-5R	5′-CCT GGC GCG TCT ACT TAG
**B. ** ***pvcrt-o***	pvcg10-1F	5′-CGC TGT CGAAGA GCC
	pvcg10-1R	5′-AGT TTC CCT CTA CAC CCG
	pvcg10-2F	5′-CGC CCG GTA GAA GC
	pvcg10-2R	5′-GGT GAG GCG ACA TGG
	pvcg10-3F	5′-GCT AAG GGC ACA TTT CC
	pvcg10-3R	5′-GTA GTC CTC AAA AGA CAC ACA TC
	pvcg10-4Fa	5′-TAT GAA GCA AAT CGC AAC AA
	pvcg10-4Fb	5′-CTT GAG AGT AAG GCA GCG AA
	pvcg10-4R	5′-TCA TCC AGA GAG CAA ACT TTC TA
**C. ** ***pvmdr1*** ** 976**	Pvmdr976 F	5′-GGA TAG TCA TGC CCC AGG ATT G
	Pvmdr976 R	5′-CAT CAA CTT CCC GGC GTA GC
	pvmdr976 internal Internal	5′-CGG CTG TAC TGA CCG GAA CGT A
**D. ** ***pvmdr1*** ** copy number**	Pvmdr F	5′-CTG ATA CAA GTG AGG AAG AAC TAC G
	pvmdrR	5′-GTC CAC CTG ACA ACT TAG ATG C
	pvaldo F	5′-GAC AGT GCC ACC ATC CTT ACC
	pvaldoR	5′-CCT TCT CAA CAT TCT CCT TCT TTC C

List of primers and their sequences used to amplify and sequence *pvmdr1* (A), *pvcrt-o* (B) and identification of the pvmdr1 Y976F mutation (C). Primers used to amplify the fragments of the *pvmdr1* and *P. vivax* aldolase reference gene in the *pvmdr1* copy number assay (D).

### Determining *pvmdr1* copy number

*Pvmdr1* gene copy number was estimated by a novel quantitative real time SybrGreen PCR assay using the Mx4000 Multiplex Quantitative PCR system (Stratagene). A single copy gene coding for *P. vivax* aldolase (GenBank Acc. No. AF247063), was used as a reference (normaliser) gene for estimating the *pvmdr1* copy number. Primers rt-pvmdrF, rt-pvmdrR, rt-pvaldoF and rt-pvaldoR, listed in Table 1, were used to amplify fragments of the *pvmdr1* or the aldolase genes respectively.

Two plasmids, containing cloned fragments of the *P. vivax* aldolase gene and either one or two copies of the *pvmdr1* fragment, were constructed de-novo and used as positive controls in every experiment. PCR reactions were performed in triplicates or quadruplets and contained 1× AB gene ABsolute™ QPCR SYBR® Green Mix (Cat. N AB-1166/a), 100 nM of ROX dye (passive reference dye), 1 µl of DNA template and 75 nM of each primer in a final volume of 25 µl. Cycling conditions were: 95°C for 15 min; followed by 40 cycles of 95°C 30 sec, 60°C 1 min and 72°C 30 sec. Fluorescence data was collected at the end of the annealing and extension steps 3 times at each and averaged. Following the amplification cycles, a melting curve analysis was performed to confirm that the correct products were synthesised. The text report, containing the threshold cycle (Ct) values for every well was exported into the Excel program (Microsoft^©^)and analysed.

The assay was optimised to achieve equal amplification efficiencies for the *pvmdr1* and aldolase gene fragments within the range of DNA concentrations from 100 ng/µl to 10 pg/µl, thus the ΔΔCt method (Applied Biosystems User Bulletin N2 (P/N 4303859B) could be used and the *pfmdr1* copy number (N) was calculated as follows: N = 2^ΔΔCt±SD^, where ΔΔCt = (Ct*_pvaldo_*-Ct*_pvmdr1_* )-(Ct *_pvaldo_*_ cal_-Ct*_pvmdr1_*_ cal_). The Ct*_pvaldo_* and Ct*_pvmdr1_* used above are threshold cycle values for the *pvmdr1* and aldolase gene respectively, whereas Ct_cal _is an average difference between Ct_aldo_ and Ctpvmdr1 obtained for the positive control containing a single copy of *pvmdr1* and aldolase gene fragments. The SD is a standard deviation calculated as follows: SD = √(S^2^ *_pvmdr1_*+S^2^ *_pvaldo_*+S^2^ *_cal_*) where S*_pvmdr1_* and *S_pvaldo_* are the standard deviations from the average Ct calculated for 3 or 4 replicates in the *pvmdr1* and *pvaldo* amplifications and S*_cal_* is an average standard deviation of the ΔCt values for the calibrator. Assessment of copy number was repeated at least twice for all isolates and the repeatability coefficient determined as 0.30 (viz 95% of repeated estimates of *pvmdr1* copy number were within 0.15 of the first).

### Data and sequence analysis

Analysis was performed using SPSS vs 14 for Windows (SPSS Inc, Chicago, Illinois, USA). The Mann-Whitney U test and Wilcoxon Signed-Rank test method was used for nonparametric comparisons, and Student's t-test (paired and unpaired) or one-way analysis of variance for parametric comparisons. Proportions were examined using χ^2^ with Yates' correction or by Fisher's exact test.

A linear regression analysis was used to determine the relationship between the log-transformed chloroquine *in vitro* susceptibility and country or genotype after correcting for duration of assay and initial percentage of parasites at ring stages, both previously shown to be confounding factors for *in vitro* susceptibility (unpublished data). In Papua, Indonesia the recurrence rate of *P. vivax* by day 28 following chloroquine monotherapy in 2004 was 65% [10]. We therefore defined, *a priori,* the clinically appropriate cut off for the IC50 as the 35^th^ percentile of isolates from this region.

Sequences were aligned using the Gap4 program, version 4.10 freely available from http://www.mrc-lmb.cam.ac.uk/pubseq/manual/gap4_windows_2.html).To investigate the relatedness of the sequences a Clustal C program was used to create the phylogenetic trees for *pvama1*, and synonymous SNPs in *pvmdr1* and *pvcrt-o* (ANGIS, http://www.angis.org.au). Unique DNA sequences described in this paper have been deposited in the GenBank under the accession No. EF458622 to EF458625.

## Results

### In vitro chloroquine susceptibility of Indonesian and Thai Isolates

**Figure 1:**
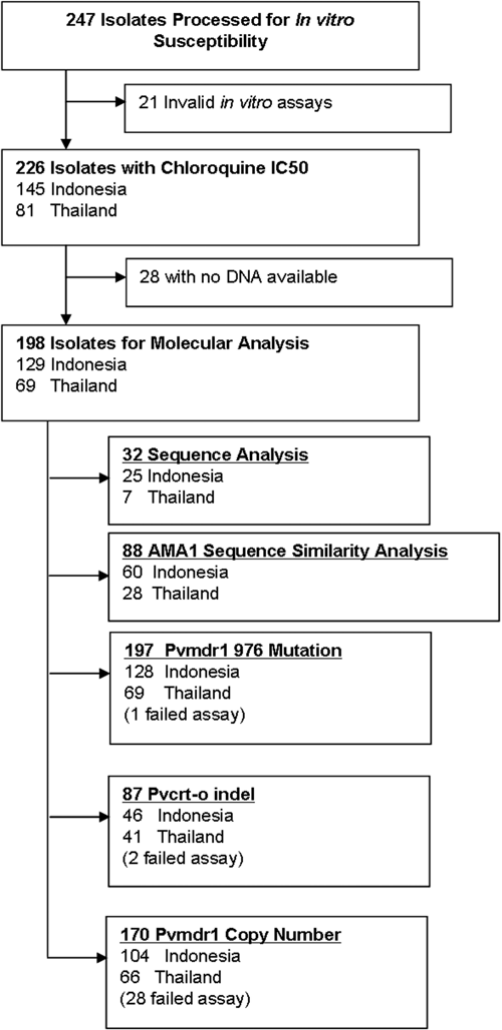
The selection of samples analysed.

Between April 2003 and December 2006, 247 isolates were assayed for *in vitro* susceptibility of which acceptable chloroquine susceptibility data could be derived in 226 (91%). Further analysis was restricted to these isolates (145 from Indonesia and 81 from Thailand); see figure 1. In total 51% (74/145) of Indonesian isolates began the assay with more than 40% ring stages prior to culture, compared to 81% (65/81) of isolates from Thailand; p<0.001. The time to reach 40% schizonts, and thus the duration of the assay, was significantly shorter in Indonesian isolates (26 hours [Range: 22–48]) compared to 36 hours [Range: 21–48] for isolates from Thailand; p<0.001.

**Figure 2:**
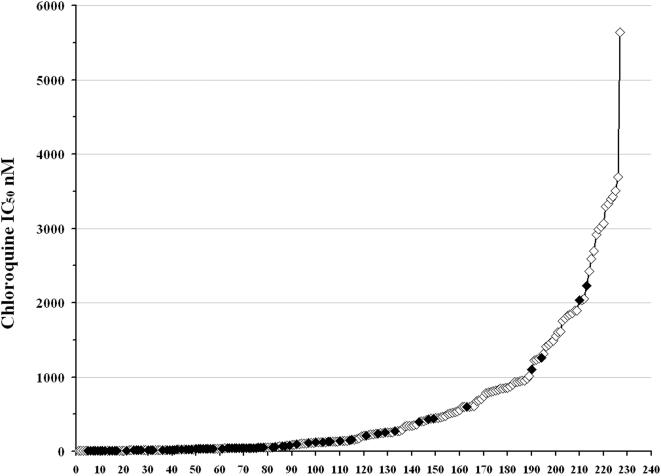
Distribution of isolate chloroquine IC_50_. 81 Thai (closed diamonds) and 141 Indonesian (open diamonds) *P. vivax* isolates ranked in order of increasing chloroquine IC_50_.

The geometric mean chloroquine IC_50_ for *P. vivax* isolates from Indonesia was 312 nM [95%Confidence Intervals CI: 237–411 nM] significantly higher than that for Thai isolates (46.8 nM [95%CI: 34.7–63.1 nM]); *p*<0.001. After ranking the 226 *P. vivax* isolates in order of decreasing chloroquine susceptibility, a continuous non-linear curve of chloroquine IC_50 _was observed (Figure 2). The 35^th^ percentile for chloroquine IC_50_ in Indonesian isolates was 220 nM. Using this as an *a priori* cut-off for clinically relevant chloroquine resistance, 13.6% (11/81) of Thai isolates were classified as resistant *in vitro*.

The chloroquine IC_50_ values were negatively correlated with both the duration of assay and the percentage of parasites at ring stage prior to culture (r_s_ = −0.576 and r_s_ = −0.496 respectively; p<0.001). However, the difference in chloroquine IC_50_ between countries remained after controlling for these factors independently (Table 2) and in a multivariate comparison; p<0.001.

**Table 2:** *In vitro* chloroquine sensitivity (nM) of isolates from Thailand and Indonesia.

		N	Geometric Mean IC_50_	95% Confidence Intervals	Range	P
**ALL**	**Indonesia**	145	312	237–411	4.6–5637	P<0.001
	**Thailand**	81	46.8	34.7–63.1	6.7–2231	
**>30 Hours Duration**	**Indonesia**	48	113	67.9–188	4.6–3024	P<0.001
	**Thailand**	69	33.2	26.0–42.5	6.7–430	
**>40% parasites at ring stage prior to culture**	**Indonesia**	74	208	139–312	4.6–3506	P<0.001
	**Thailand**	65	33.7	25.8–44.1	6.7–1264	

Values given overall and after selecting cultures with greater than 30 hour duration of assay or a starting with more 40% of parasites at ring stage. Criteria for duration of assay and percentage of rings in initial culture taken from Tasanor et al 2002 [19]

In total 16 Indonesian isolates were assayed for chloroquine with and without 0.9 µM verapamil. The median IC_50_ was not significantly different: 258 nM [Interquartile Range: 69–950 nM] for chloroquine plus verapamil compared to 157 nM [IQR: 23–332 nM] in the chloroquine alone assay; *p* = 0.56.

### Polymorphisms in *pvmdr1*, *pvcrt-o* and *pvama1* in core samples

Using Sal1 as the reference strain, sequence analysis of 32 core isolates revealed single nucleotide polymorphisms (SNP) at 5 loci of *pvmdr1*, two non-synonymous mutations resulting in amino acid changes at Y976F and L1076F (Table 3) and three synonymous SNPs (at codons 493, 908 and 1396). In these core isolates the Y976F mutation was significantly more prevalent in Indonesian isolates (96%, 24/25) compared to Thai isolates (43%, 3/7); *p* = 0.004.

**Table 3:** Mutations in *pvmdr1* and *pvcrt-o* among 32 core *Plasmodium vivax* isolates from Indonesian and Thailand and the reference strains SAL1.

Sample	Origin	Genotype groups				Polymorphisms in *pvmdr1* and *pvcrt-0*				
						*pvmdr1*			*pvcrt-0* exon	
		Msp1 LP	Msp3 RFLP	AMA1 Sequence	Combined Genotype	Copy number	Y976F SNP	F1076L SNP	K10 Insert	I43M SNP
**SAL 1**	**Central America**	a	a	a	**a**	1	Y	F	-	I
**ANV20**	**Indonesia**	b	a	b	**b**	1	**F**	**L**	-	I
**VI21**	**Thailand**	a	b	c	**c**	1	Y	**L**	**K**	I
**VI20**	**Thailand**	a	a	d	**d**	1	**F**	**L**	**K**	I
**VI32**	**Thailand**	a	a	d	**d**	**2**	Y	**L**	**K**	I
**VI5**	**Thailand**	a	a	c	**e**	**2**	Y	**L**	**K**	I
**VRP21**	**Indonesia**	b	a	e	**f**	1	**F**	**L**	-	I
**VI1**	**Thailand**	a	a	f	**g**	1	**F**	**L**	-	I
**PV14**	**Thailand**	b	c	d	**h**	1	**F**	**L**	**K**	I
**VI23**	**Thailand**	a	a	g	**i**	**2**	Y	**L**	**K**	I
**VP63**	**Indonesia**	b	a	h	**j**	1	**F**	**L**	-	I
**FC1010**	**Indonesia**	b	a	i	**k**	1	**F**	**L**	-	I
**UVT27**	**Indonesia**	b	a	i	**k**	1	**F**	**L**	-	I
**FC1232**	**Indonesia**	b	a	c	**l**	1	**F**	**L**	-	I
**VP56**	**Indonesia**	b	a	c	**l**	1	**F**	**L**	-	I
**FC1108**	**Indonesia**	b	a	c	**l**	1	**F**	**L**	-	I
**VRP23**	**Indonesia**	b	a	c	**l**	1	**F**	**L**	-	I
**VP59**	**Indonesia**	a	a	c	**m**	1	**F**	**L**	-	I
**FC1158**	**Indonesia**	a	a	c	**m**	1	**F**	**L**	-	I
**ANV15**	**Indonesia**	b	d	c	**n**	1	**F**	**L**	-	I
**FC1248**	**Indonesia**	b	d	c	**n**	1	**F**	**L**	-	I
**VRP20**	**Indonesia**	a	c	j	**o**	1	**F**	**L**	-	I
**UVT22**	**Indonesia**	a	a	i	**p**	1	Y	**L**	-	I
**ANV16**	**Indonesia**	c	a	i	**q**	1	**F**	**L**	-	I
**FC1083**	**Indonesia**	a	c	k	**r**	1	**F**	**L**	-	I
**UVT44**	**Indonesia**	a	a	b	**s**	1	**F**	**L**	-	I
**ANV18**	**Indonesia**	c	a	i	**t**	1	**F**	**L**	-	I
**ANV12**	**Indonesia**	c	d	l	**u**	1	**F**	**L**	-	I
**UVT70**	**Indonesia**	c	a	m	**v**	1	**F**	**L**	-	I
**FC269**	**Indonesia**	b	d	n	**w**	1	**F**	**L**	-	I
**FC1290**	**Indonesia**	a	a	o	**x**	1	**F**	**L**	-	I
**FC10122**	**Indonesia**	b	a	l	**y**	1	**F**	**L**	-	I

Isolates are grouped and ordered alphabetically according to the combined *pvmsp1*, *pvmsp3* and *ama1* genotype. SNP positions and corresponding amino acid changes relative to the SAL1 reference strain are in bold.

One insert and one non-synonymous SNP were found in *pvcrt-o* exons, the most prevalent of which was the insertion of the trinucleotide AAG, coding for amino acid Lysine (K) at amino acid position 10 in the first exon (see table 3). The insert was found in 86% (6/7) of Thai isolates and 0% (0/25) Indonesian isolates; *p*<0.001.

**Figure 3:**
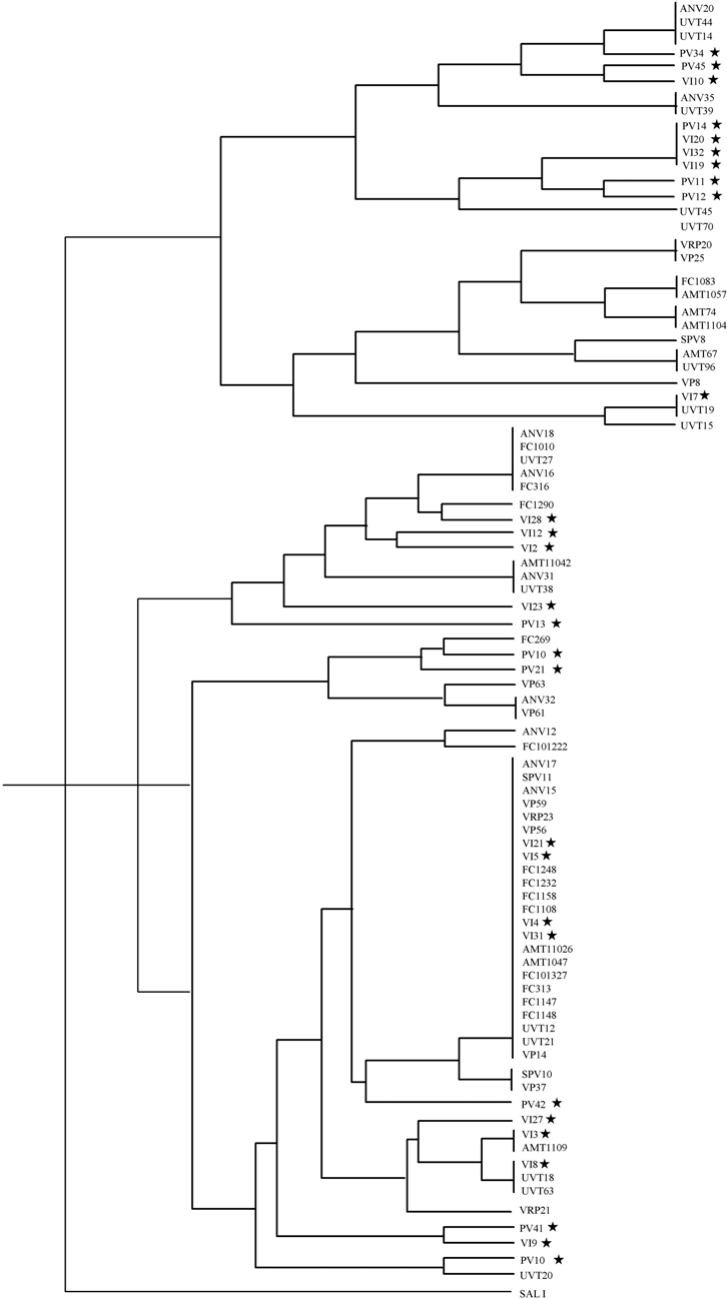
Sequence relatedness among *P. vivax* isolates from different locations according to clustal-c analysis of *pvama1* sequence. Stars indicate Thai isolates.

Analysis of three loci (*pvama1, pvmsp1 and pvmsp3*) revealed 26 combined haplotypes in the 32 core isolates indicating that the isolates are of a diverse genetic background. Clustal-c analysis of partial *pvama1* sequences from 89 isolates (Figure 3) showed no evidence that sequence diversity was less among isolates from within each field site compared to between locations.

### *P. vivax* chloroquine susceptibility and *pvmdr1* and *pvcrt-o* polymorphisms

**Figure 4:**
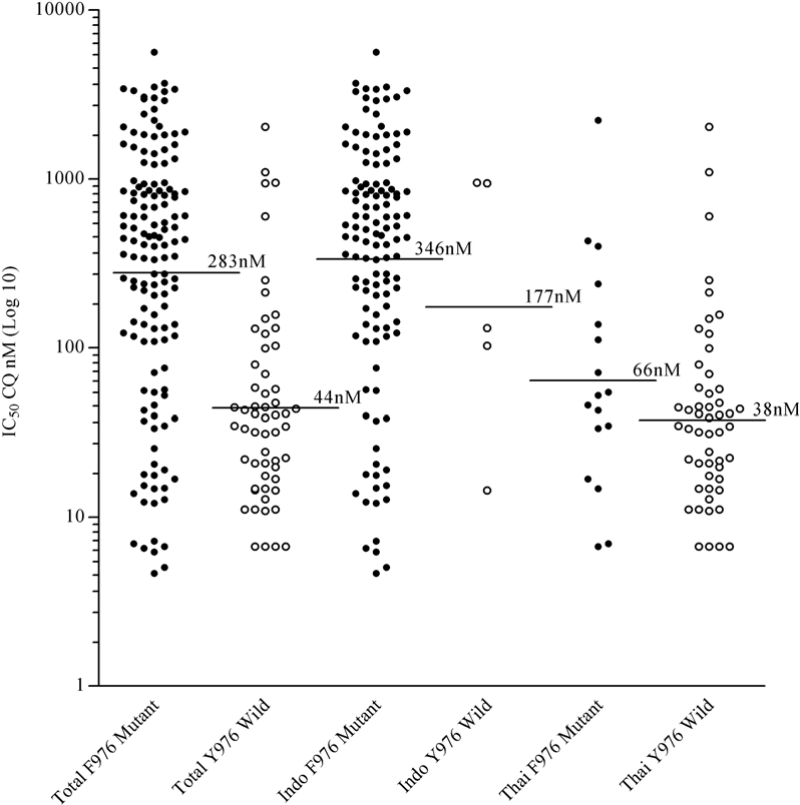
Association between *P. vivax* chloroquine IC_50_ and Y976F mutations in *pvmdr1.* The solid black horizontal lines show the geometric mean IC_50_ (nM) of the *P. vivax* population samples.

Polymorphisms were assessed for the *pvmdr1* SNP at codon 976 in an additional 165 isolates with *in vitro* susceptibility data and for the *pvcrt-o* insertion at amino acid position 10 in an additional 55 isolates (Figure 1). After combining these with the core isolates the Y976F allele was found in 96.1% (123/128) of Indonesian isolates compared to 25% (17/69) of Thai isolates (*p*<0.001). Overall, the geometric mean chloroquine IC_50_ in isolates with the Y976F mutation was 283 nM [95%CI: 211–379], significantly higher than that in isolates with the wild type allele (geometric mean = 44.5 nM [95%CI: 31.3–63.4]; *p*< 0.001). In Thailand, isolates with the Y976F mutation had a mean IC_50_ of 65.6 nM [95%CI: 29.9–144] compared to 39.0 nM [95%CI: 27.8–54.8] in those with the wild type allele (*p* = 0.008, after controlling for assay duration and percentage of rings pre incubation). The trend was similar in Indonesian isolates, however the proportion of Y976F mutation almost reached fixation and thus prohibited analysis of the correlation between the polymorphism and the phenotype (see figure 4).

The *pvcrt-o* AAG insertion occurred in 76% (31/41) of Thai isolates, but only 2.2% (1/46) of the Indonesian isolates (*p*<0.001). Overall the *pvcrt-o* AAG insertion was associated with a significant reduction in chloroquine IC_50_, (geometric mean 47.6 nM [95%CI: 29.7–76.1] vs 261 nM [95%CI: 172–396]; *p*<0.001). After stratifying by geographical location, the AAG insertion of *pvcrt-o* was not linked to the *pvmdr1* Y976F mutation and was not significantly associated with reduced chloroquine IC_50_ **.**

### *P. vivax* chloroquine susceptibility and *pvmdr1* copy number

The *pvmdr1* copy number was successfully quantified in 86% (170/198) of isolates tested. In total 23% (15/66) of isolates from Thailand had an increased *pvmdr1* copy number (13 with 2 copies and two with 3 copies) and none (0/104) from Indonesia; p<0.001. In Thailand all (15/15) of the isolates with increased copy number were wild type at 976, compared to 67% (34/51) of those with a single copy number of *pvmdr1* (p = 0.007). Although isolates with increased *pvmdr1* copy number had significantly lower chloroquine IC_50_s (geometric mean = 39.6 [95%CI: 24.5–64.1]) compared to isolates with single copies of *pvmdr1* (geometric mean = 184 [95%CI: 137–247]; p<0.001); this was not apparent after stratification by country.

## Discussion

In clinical studies, *P. vivax* remains predominantly sensitive to chloroquine in Thailand, whereas in Papua, Indonesia high grade clinical resistance is already established [10], [25], [26]. In 2004 a chemotherapeutic study at the Papuan field site demonstrated that 65% of patients failed treatment within 28 days of chloroquine monotherapy, 16% of whom had early high grade failures. Treatment guidelines were changed accordingly to an Artemisinin combination therapy for both *P. falciparum* and *P. vivax* [31], precluding further clinical studies on the use of chloroquine monotherapy in this region. In the present study we have continued our analysis of chloroquine resistance *P. vivax* using an identical *in vitro* methodology in both Indonesia and Thailand and correlating our results with the known data on the clinical efficacy of chloroquine in these regions. The Indonesian *P. vivax* isolates tested had a significantly higher median chloroquine IC_50_ and a higher proportion above the resistance threshold compared with that of Thai isolates.

The determination of chloroquine susceptibility in *P. vivax* using the schizont maturation method is more complicated than the same method in *P. falciparum*, due to the asynchrony of the vivax parasites and possible differential responses to the drug by parasites at different development stages. Patient samples with higher percentage of trophozoites and late rings require less incubation time to reach maturation (unpublished data). The decreased susceptibility to chloroquine in these samples provides a plausible explanation for our observation of the negative correlations between IC_50_ and culture duration as well as with percentage of rings at the start of culture. Although differentiating between these possibilities is difficult, we attempted to control for these confounding factors by stratifying our results according to culture duration and the percentage of rings at the start of culture; the differences in IC_50_ between isolates from Indonesia and Thailand remained.

The *in vitro* cut-off defining clinically relevant chloroquine resistance has yet to be defined. Using the clinical failure rate (65%) observed in the same area, we defined this from the 35^th^ percentile as 220 nM, almost double the 100nM cut-off value for chloroquine resistance in *P. falciparum.* However clinical failures may have included some relapses that occur within the 28 day follow up period, and the true rate of recrudescence maybe lower. Hence this threshold is likely to be the minimum value associated with resistance.

The chloroquine IC_50_ of Thai isolates were significantly lower than the Indonesian isolates (Geometric mean = 46.8 vs 312 nM), although the difference was less after controlling for the duration of assay (33.2 vs 113 nM) or initial stage of parasite prior to culture (33.7 vs 208 nM). Interestingly 13.6% (11/81) of Thai isolates had a chloroquine IC_50_ over 220nM. Although clinical studies in Thailand in the 1990s have repeatedly demonstrated the continued efficacy of chloroquine monotherapy for *P. vivax* [26], [27], our *in vitro* results raise the possibility that clinically relevant chloroquine resistance may now be present at low prevalence along the western border of Thailand. This is corroborated by a recent clinical study from the Thai-Myanmar border demonstrating 34% *P. vivax* recurrence rates within 28 days of chloroquine monotherapy [32].

The correlation between *in vitro* susceptibility and clinical efficacy at our two study sites validates our *in vitro* susceptibility test and suggests that the adapted schizont maturation method may be usefully applied to investigate the emergence of drug resistance in *P. vivax* in other locations. Furthermore, the ability to define parasite susceptibility free from the confounding factors of host and environment provides a useful framework from which to investigate putative molecular markers of drug resistance. We used our carefully defined sample set to test for associations between the *in vitro* response to chloroquine and polymorphisms of the orthologues of two genes (*pvmdr1* and *pvcrt-o*) known to be important determinants of chloroquine resistance in *P. falciparum*. Although previous studies have not established a link between these genes and chloroquine resistant *P. vivax* , these generally used a relatively small number of clinical isolates in which the phenotypic definition was possibly confounded by patient immunity, re-infection and relapses [22], [23]. Brega et al identified the *pvmdr1* Y976F and 1076 mutation in a small number of Thai and Indonesian isolates, although *in vitro* and clinical correlates were not presented [33].

In the present study we found two polymorphisms which were correlated with *in vitro* chloroquine susceptibility: the *pvmdr1* Y976F mutation and an insertion in the 1^st^ exon (amino acid position 10) of *pvcrt-o*. Overall both polymorphisms were associated with a significant increase in chloroquine IC_50_. In Papua, Indonesia, where the Y976F mutation has reached fixation and the AAG insertion was almost absent it was not possible to test the relevance of these markers. However in Thailand, the Y976F mutation was present in 25% (17/69) of isolates and associated with 1.7 fold increase in IC_50_ to chloroquine.

To rule out the possibility that the polymorphisms were related to geographical isolation of the samples, we performed phylogeny analyses to compare the samples from two locations on *pvama1*, a marker unrelated to chloroquine pressure. The results did not show clustering of samples with location. In addition, we analysed *pvmdr1* sequence including all synonymous changes in *pvmdr1* which are presumed not to be selected by drug pressure. Again we did not see clustering of the samples with location. These analyses suggest that the Y976F is unlikely to be geographically associated with the Papua location *per se*, and provide further evidence for its selection by chloroquine selective pressure.

Notably a small number of isolates with high IC_50_ values were observed from both sites in the absence of the 976 mutation, and vice versa, suggesting that other major molecular determinants are likely to be involved. However a role of *pvmdr1* in modulating chloroquine susceptibility is supported by the almost ubiquitous selection of the Y976F allele in Papua, where high grade chloroquine resistance is known to predominate.

Gene amplification of the *pfmdr1* gene has been shown to be a major determinant of multidrug resistance in *P. falciparum*. Furthermore on the Thai-Myanmar border widespread deployment of mefloquine has been associated with high prevalence of *P. falciparum* isolates with increased *pfmdr1* copy number and an associated decrease in susceptibility to mefloquine, quinine, lumefantrine, halofantrine and the artemisinin derivatives in *P. falciparum* [34]. In this study we report that amplification of *pvmdr1* copy number occurs in *P. vivax* in Thailand, but not Papua, where mefloquine has not been used. Our data raise the prospect of similar molecular mechanisms of multi drug resistant phenotype as found in *P. falciparum*, although further work is needed to confirm this.

In conclusion, using an *in vitro* susceptibility assay, we have been able to define a spectrum of chloroquine susceptibility in *P. vivax* and discriminate between populations with differing levels of clinical efficacy following chloroquine monotherapy. Although the molecular mechanism underlying chloroquine resistance *P. vivax* may involve multigenic loci, the *pvmdr1* polymorphism at Y976F may provide a useful tool to monitor the emergence of chloroquine resistance.

## References

[1] Mendis K, Sina BJ, Marchesini P, Carter R (2001). The neglected burden of Plasmodium vivax malaria.. Am J Trop Med Hyg.

[2] Price RN, Tjitra E, Guerra CA, Yeung S, White NJ (2007). Vivax malaria: neglected and not benign.. Am J Trop Med Hyg In Press.

[3] Hay SI, Guerra CA, Tatem AJ, Noor AM, Snow RW (2004). The global distribution and population at risk of malaria: past, present, and future.. Lancet Infect Dis.

[4] Rieckmann KH, Davis DR, Hutton DC (1989). Plasmodium vivax resistance to chloroquine?. Lancet.

[5] Baird JK, Sustriayu Nalim MF, Basri H, Masbar S, Leksana B (1996). Survey of resistance to chloroquine by Plasmodium vivax in Indonesia.. Trans R Soc Trop Med Hyg.

[6] Baird JK, Basri H, Purnomo, Bangs MJ, Subianto B (1991). Resistance to chloroquine by Plasmodium vivax in Irian Jaya, Indonesia.. Am J Trop Med Hyg.

[7] Murphy GS, Basri H, Purnomo, Andersen EM, Bangs MJ (1993). Vivax malaria resistant to treatment and prophylaxis with chloroquine.. Lancet.

[8] Tjitra E, Baker J, Suprianto S, Cheng Q, Anstey NM (2002). Therapeutic efficacies of artesunate-sulfadoxine-pyrimethamine and chloroquine-sulfadoxine-pyrimethamine in vivax malaria pilot studies: relationship to Plasmodium vivax dhfr mutations.. Antimicrob Agents Chemother.

[9] Sumawinata IW, Bernadeta, Leksana B, Sutamihardja A, Purnomo (2003). Very high risk of therapeutic failure with chloroquine for uncomplicated Plasmodium falciparum and P. vivax malaria in Indonesian Papua.. Am J Trop Med Hyg.

[10] Ratcliff A, Siswantoro H, Kenangalem E, Wuwung M, Brockman A (2007). Therapeutic response of multidrug-resistant Plasmodium falciparum and P. vivax to chloroquine and sulfadoxine-pyrimethamine in southern Papua, Indonesia.. Trans R Soc Trop Med Hyg.

[11] Fryauff DJ, Tuti S, Mardi A, Masbar S, Patipelohi R (1998). Chloroquine-resistant Plasmodium vivax in transmigration settlements of West Kalimantan, Indonesia.. Am J Trop Med Hyg.

[12] Marlar T, Myat Phone K, Aye Yu S, Khaing Khaing G, Ma S (1995). Development of resistance to chloroquine by Plasmodium vivax in Myanmar.. Trans R Soc Trop Med Hyg.

[13] Soto J, Toledo J, Gutierrez P, Luzz M, Llinas N (2001). Plasmodium vivax clinically resistant to chloroquine in Colombia.. Am J Trop Med Hyg.

[14] Phillips EJ, Keystone JS, Kain KC (1996). Failure of combined chloroquine and high-dose primaquine therapy for Plasmodium vivax malaria acquired in Guyana, South America.. Clin Infect Dis.

[15] Phan GT, de Vries PJ, Tran BQ, Le HQ, Nguyen NV (2002). Artemisinin or chloroquine for blood stage Plasmodium vivax malaria in Vietnam.. Trop Med Int Health.

[16] Kurcer MA, Simsek Z, Kurcer Z (2006). The decreasing efficacy of chloroquine in the treatment of Plasmodium vivax malaria, in Sanliurfa, south-eastern Turkey.. Ann Trop Med Parasitol.

[17] Russell BM, Udomsangpetch R, Rieckmann KH, Kotecka BM, Coleman RE (2003). Simple in vitro assay for determining the sensitivity of Plasmodium vivax isolates from fresh human blood to antimalarials in areas where P. vivax is endemic.. Antimicrob Agents Chemother.

[18] Chotivanich K, Udomsangpetch R, Chierakul W, Newton PN, Ruangveerayuth R (2004). In vitro efficacy of antimalarial drugs against Plasmodium vivax on the western border of Thailand.. Am J Trop Med Hyg.

[19] Tasanor O, Noedl H, Na-Bangchang K, Congpuong K, Sirichaisinthop J (2002). An in vitro system for assessing the sensitivity of Plasmodium vivax to chloroquine.. Acta Trop.

[20] Fidock DA, Nomura T, Talley AK, Cooper RA, Dzekunov SM (2000). Mutations in the P. falciparum digestive vacuole transmembrane protein PfCRT and evidence for their role in chloroquine resistance.. Mol Cell.

[21] Reed MB, Saliba KJ, Caruana SR, Kirk K, Cowman AF (2000). Pgh1 modulates sensitivity and resistance to multiple antimalarials in Plasmodium falciparum.. Nature.

[22] Nomura T, Carlton JM, Baird JK, del Portillo HA, Fryauff DJ (2001). Evidence for different mechanisms of chloroquine resistance in 2 Plasmodium species that cause human malaria.. J Infect Dis.

[23] Sa JM, Nomura T, Neves J, Baird JK, Wellems TE (2005). Plasmodium vivax: allele variants of the mdr1 gene do not associate with chloroquine resistance among isolates from Brazil, Papua, and monkey-adapted strains.. Exp Parasitol.

[24] Sa JM, Yamamoto MM, Fernandez-Becerra C, de Azevedo MF, Papakrivos J (2006). Expression and function of pvcrt-o, a Plasmodium vivax ortholog of pfcrt, in Plasmodium falciparum and Dictyostelium discoideum.. Mol Biochem Parasitol.

[25] Baird JK (2004). Chloroquine resistance in Plasmodium vivax.. Antimicrob Agents Chemother.

[26] Looareesuwan S, Wilairatana P, Krudsood S, Treeprasertsuk S, Singhasivanon P (1999). Chloroquine sensitivity of Plasmodium vivax in Thailand.. Ann Trop Med Parasitol.

[27] Luxemburger C, van Vugt M, Jonathan S, McGready R, Looareesuwan S (1999). Treatment of vivax malaria on the western border of Thailand.. Trans R Soc Trop Med Hyg.

[28] Padley D, Moody AH, Chiodini PL, Saldanha J (2003). Use of a rapid, single-round, multiplex PCR to detect malarial parasites and identify the species present.. Ann Trop Med Parasitol.

[29] Figtree M, Pasay CJ, Slade R, Cheng Q, Cloonan N (2000). Plasmodium vivax synonymous substitution frequencies, evolution and population structure deduced from diversity in AMA 1 and MSP 1 genes.. Mol Biochem Parasitol.

[30] Bruce MC, Galinski MR, Barnwell JW, Snounou G, Day KP (1999). Polymorphism at the merozoite surface protein-3alpha locus of Plasmodium vivax: global and local diversity.. Am J Trop Med Hyg.

[31] Ratcliff A, Siswantoro H, Kenangalem E, Maristela R, Wuwung RM (2007). Two fixed-dose artemisinin combinations for drug-resistant falciparum and vivax malaria in Papua, Indonesia: an open-label randomised comparison.. Lancet.

[32] Guthmann JP, Pittet A, Lesage A, Imwong M, Lindegardh N (2007). Plasmodium vivax resistance to chloroquine in Dawei, southern Myanmar.. Trop Med Int Health. In Press.

[33] Brega S, Meslin B, de Monbrison F, Severini C, Gradoni L (2005). Identification of the Plasmodium vivax mdr-like gene (pvmdr1) and analysis of single-nucleotide polymorphisms among isolates from different areas of endemicity.. J Infect Dis.

[34] Price RN, Cassar C, Brockman A, Duraisingh M, van Vugt M (1999). The pfmdr1 gene is associated with a multidrug-resistant phenotype in Plasmodium falciparum from the western border of Thailand.. Antimicrob Agents Chemother.

